# Cluster Subcutaneous Allergen Specific Immunotherapy for the Treatment of Allergic Rhinitis: A Systematic Review and Meta-Analysis

**DOI:** 10.1371/journal.pone.0086529

**Published:** 2014-01-28

**Authors:** Shaoyan Feng, Ying Xu, Renqiang Ma, Yueqi Sun, Xi Luo, Huabin Li

**Affiliations:** 1 Allergy and Cancer Center, Otorhinolarygology Hospital, The First Affiliated Hospital of Sun Yat-sen University, Guangzhou, China; 2 Department of Otolaryngology, The Fifth Affiliated Hospital of Sun Yat-sen University, Zhuhai, China; 3 Department of Epidemiology and Biostatistics, School of Public Health, Guangdong Pharmaceutical University, Guangzhou, China; University of York, United Kingdom

## Abstract

**Background:**

Although allergen specific immunotherapy (SIT) represents the only immune- modifying and curative option available for patients with allergic rhinitis (AR), the optimal schedule for specific subcutaneous immunotherapy (SCIT) is still unknown. The objective of this study is to systematically assess the efficacy and safety of cluster SCIT for patients with AR.

**Methods:**

By searching PubMed, EMBASE and the Cochrane clinical trials database from 1980 through May 10th, 2013, we collected and analyzed the randomized controlled trials (RCTs) of cluster SCIT to assess its efficacy and safety.

**Results:**

Eight trials involving 567 participants were included in this systematic review. Our meta-analysis showed that cluster SCIT have similar effect in reduction of both rhinitis symptoms and the requirement for anti-allergic medication compared with conventional SCIT, but when comparing cluster SCIT with placebo, no statistic significance were found in reduction of symptom scores or medication scores. Some caution is required in this interpretation as there was significant heterogeneity between studies. Data relating to Rhinoconjunctivitis Quality of Life Questionnaire (RQLQ) in 3 included studies were analyzed, which consistently point to the efficacy of cluster SCIT in improving quality of life compared to placebo. To assess the safety of cluster SCIT, meta-analysis showed that no differences existed in the incidence of either local adverse reaction or systemic adverse reaction between the cluster group and control group.

**Conclusion:**

Based on the current limited evidence, we still could not conclude affirmatively that cluster SCIT was a safe and efficacious option for the treatment of AR patients. Further large-scale, well-designed RCTs on this topic are still needed.

## Introduction

Allergic rhinitis (AR) is a common airway disease with a reported prevalence of 10–30%. Although AR is not a serious illness, it is clinically relevant because it underlies many complications (eg. asthma) and affects quality of life and productivity at work or school. Current treatment modalities include allergen avoidance, antihistamine, nasal steroid and allergen specific immunotherapy (SIT) [1]. Comparing to the symptom-releasing options (eg. antihistamine and nasal steroid), SIT (subcutaneous or sublingual route) represents the only immune-modifying and curative available option for the treatment of AR patients [2]. In contrast to the sublingual SIT, subcutaneous immunotherapy (SCIT) entails repeated injections with allergen extracts. Novel data demonstrate the efficacy of SCIT also as a preventive strategy to reduce onset of new sensitization to non-related allergens, progression from AR to asthma, and to improve long-term outcome of already established asthma in addition to acting as a therapeutic agent. However, despite the well-established benefits of SCIT, only a small percentage of candidate AR patients were willing to accept this therapeutic option with good compliance [3].

Inconvenience is one of the primary reasons for discontinuation of SCIT [4]. In the conventional build-up schedule, the frequency of injections generally ranges from 1 to 2 times per week, with a single injection given each visit. The duration of the build-up phase depends on the frequency of the injections but generally ranges from 3 to 6 months. To avoid the disadvantages of conventional schedule of SCIT, it is necessary to design administration schedules that shorten the build-up phase without increasing the adverse reactions rate [5], [6]. Rush schedule is performed administering a few increasing dosages during the same day and for a few consecutive days, until the maximum tolerated dose is reached. This schedule involves nonetheless higher risks for adverse events for patients. Their use is therefore limited to hospitalized patients under careful monitoring with the supervision of adequately trained personnel. As an alternative, cluster SCIT as another accelerated schedule has been suggested by entailing administering several injections at increasing doses (generally 2 to 3 per visit) sequentially in a single day of treatment on nonconsecutive days [7], [8]. The maintenance dose is generally achieved within 4 to 8 weeks. Up to now, the efficacy and safety of cluster SCIT in clinical practice is yet not to be determined.

## Materials and Methods

### Search Strategy

We searched PubMed, EMBASE and the Cochrane clinical trials database from 1980 through May 10th, 2013. The search strategies used the following major keywords: “allergic rhinitis”, “immunotherapy”and“cluster”. Bibliographies of all potentially relevant retrieved studies, identified relevant articles (including unpublished and meta-analysis studies, a follow-up from reference lists of relevant articles and personal contact with experts in this field) and international guidelines were searched by hand.

The following inclusive selection criteria included: (i) population: patients with diagnosed AR with or without asthma; (ii) intervention: cluster SCIT; (iii) comparison intervention: conventional SCIT or placebo with or without other pharmacological treatments; and (iv) outcome measures: rhinitis symptom scores, medication scores, overall quality of life, or adverse events; and (v) study design: randomized controlled trial (RCT).

### Data Extraction

Two reviewers independently screened studies for inclusion, retrieved potentially relevant studies, and determined study eligibility. For each study, we recorded the first author, year of publication, study type, number and description of subjects, type of allergen, number of injection per visit in build-up phase and final dose reached, total duration and measurement method of outcomes. Any disagreements were resolved by discussion and consensus. A third investigator was consulted in case of disagreement to improve accuracy. The analytical data missing from the primary reports were requested from their authors.

### Quality Assessment

The methodological quality of each RCT was assessed according to the Cochrane Collaboration’s tool for assessing risk of bias [9].

### Statistical Analysis

Differences were expressed as risk difference (RD), risk ratio(RR), or weighted mean difference(WMD) with 95% confidence intervals (CIs) for dichotomous outcomes and continuous outcomes, respectively. Statistical heterogeneity across trials was assessed with the χ^2^ statistic (p<0.1) and the *I* ^2^ statistic [10]. The *I* ^2^ statistic measures the proportion of overall variation that is attributable to between-study heterogeneity. As a guide, *I* ^2^ values of 25%, 50%, and 75% correspond to low, medium, and high levels of heterogeneity [11]. For the χ^2^ statistic, the heterogeneity test was considered statistically significant if the p-value was under 0.1. When a significant heterogeneity was found, a random-effects model was used to calculate the pooled results and 95% confidence interval as well as prediction intervals around the mean were reported. Otherwise, a fixed-effects model was applied. If considerable variation was noted among studies, a brief qualitative analysis of evidence was presented. Publication bias was assessed by visually inspecting funnel plots [12]. A P value of less than 0.05 was considered statistically significant. All statistical analyses were performed using Revman 5.1.0 (The Cochrane Collaboration, Oxford, UK).

## Results

### Study Identification and Selection

**Figure 1:**
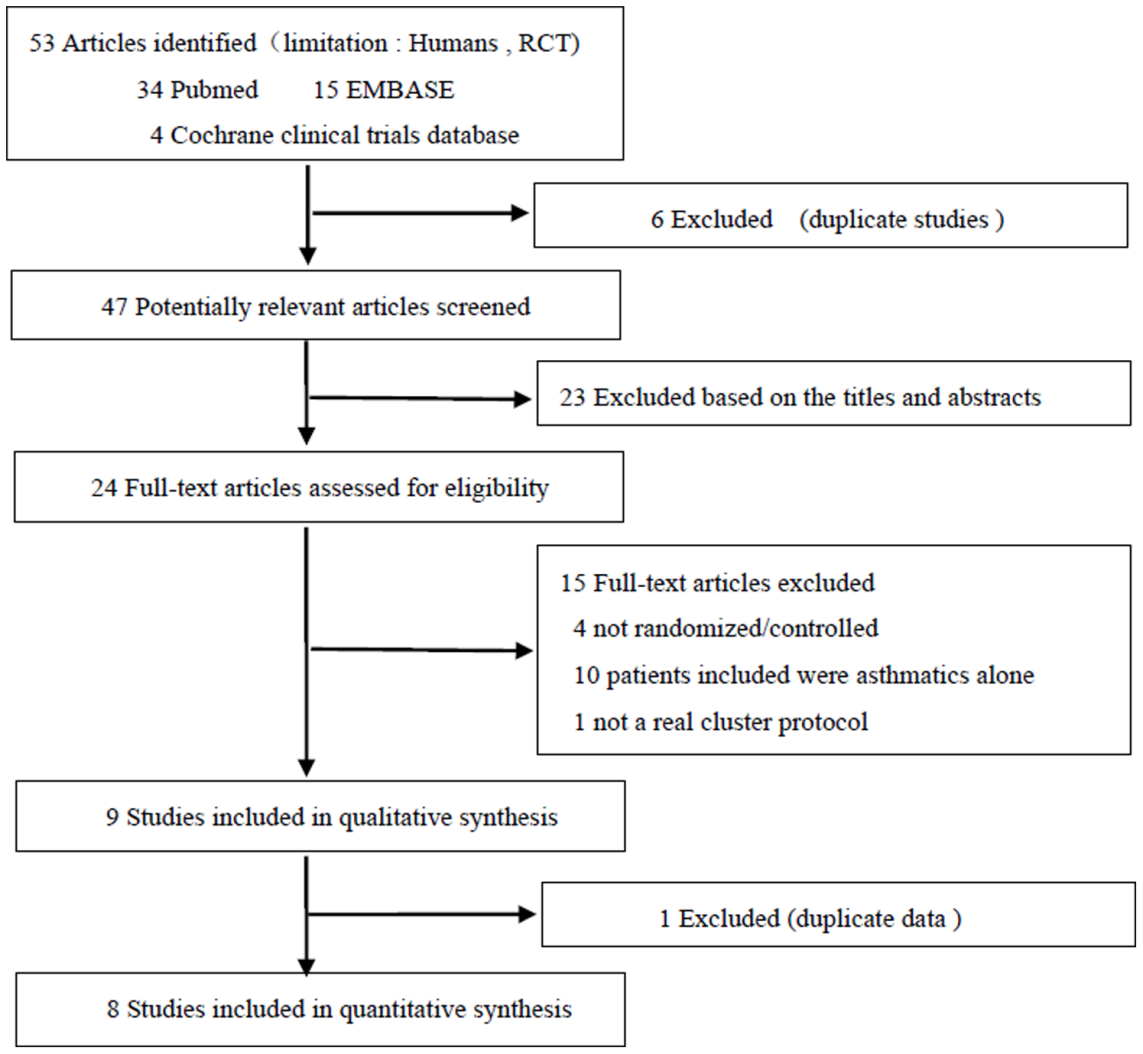
Flowchart of studies included. RCT, randomized controlled trial.

An initial database search identified a total of 53 RCTs. Six RCTs were excluded because of duplicate studies, and 23 RCTs were excluded based on the titles and abstracts. The remaining 24 full-text articles were reviewed for more detailed evaluation; 15 of them were also excluded. Of them, 4 studies were not real randomized/controlled studies [13]–[16], 10 studies in which some patients included were asthmatics alone without AR [17]–[26], and 1 study did not perform a real cluster protocol [27]. Additional, a RCT was excluded due to repeated data [28]. Finally, 8 RCTs that met our inclusion criteria were included in the present analysis [29]–[36]. The flowchart of studies included is shown in Figure 1.

### Characteristics of the Studies

The main characteristics of the included studies are listed in Table 1. In total there were 567 participants: 310 as investigated and 257 as control. In the build-up phase the number of injections received by each individual ranged from 5 to 16. The types of vaccines used in six studies were extracts, and remaining two were allergoids. Six studies compared cluster SCIT with placebo [29]–[31], [33], [34], [36]. However, Tabar et al. [32] and Zhang et al. [35] compared cluster SCIT with conventional SCIT. Seven of the included RCTs adopted a 2-armed parallel group design [29], [30], [32]–[36], one had a 4-armed parallel group design [31]. In Nanda et al.’ study [31], 28 patients with cat allergy randomized in a double-blind study were assigned to one of 4 treatment groups: placebo or cat hair and dander extract containing 0.6 mg of Fel d 1, 3 mg of Fel d 1, and 15 mg of Fel d 1 at maintenance. Four RCTs tested cluster SCIT for treating AR [31], [32], [35], [36], and 4 RCTs tested cluster SCIT for preventing AR [29], [30], [33], [34]. In the 4 prevention trials, patients were classified as having seasonal AR(SAR). Before onset of the season, patients received cluster SCIT. Symptoms were then evaluated during the following days.

**Table 1:** Characteristics of included studies.

Study	Group(N)	Intervention Protocol	VaccineType	Age	Build-upphase	No. of injections pervisit in build-up phase	Final dose	Total duration	Outcome Measure
Walker2001	I (22)	Grass pollen	Extract	32(22–64)	4W	3/2/2/1/1/1/1	20 µg	2 Y	NSS/MS/HRQL/CT/S
	C (22)	Placebo		32(23–59)	4W	3/2/2/1/1/1/1		2 Y	
Crimi2004	I(15)	Parietaria judaica	Extract	32(21–54)	7W	2/2/2/2/1/1/1/1	4.8 µg	3Y	NSS/MS/S
	C(15)	Placebo		34(20–53)	7W	2/2/2/2/1/1/1/1		3Y	
Nanda2004	I(20)	Cat hair and dander	Extract	>18	5W	2/2/2/2/1	0.6/3/15 µg	1Y	TNC/CT/IgG4/IgE/S
	C(6)	Placebo		>18	5W	2/2/2/2/1		1Y	
Tabar2005	I (120)	Der p	Extract	19.34±9.8	6 W	4/3/2/2/2/2/1	10.12 µg	1 Y	NSS/MS/CT/IgG4/IgE/S
	C (119)	Der p and Placebo	Extract	18.47±9.49	12 W	4/3/2/2/2/2/1	9.06 µg	1 Y	
Colás2006	I (41)	Sal k	Allergoid	34(18–51)	2 W	3/3/1	22.5 µg	1 Y	NSS/MS/CT/HRQL/S
	C (19)	Placebo		33(18–51)	2 W	3/3/1		1 Y	
Subiza2008	I (22)	D.G and T.P	Allergoid	29.7(12–60)	2W	2/2/1	12.3 µg	2.3M	TNC/CT/S
	C (11)	No treatment		30.8(12–60)		0		2.8M	
Zhang2009	I (45)	Der p	Extract	25(15–36)	6 W	3/2/2/2/2/2/1	9.8 µg	1 Y	NSS/MS/CT/IgE/HRQL/S
	C (44)	Der p	Extract	25(12–34)	14 W	1 inj. Weekly	9.8 µg	1 Y	
Lou2012	I (25)	Der p and medicine	Extract	12 (9–13)	6 W	3/2/2/2/2/2/1	9.8 µg	1 Y	NSS/MS/T cells/Cytokine/IgG4/IgE
	C (21)	Placebo		11 (8–13)		0		1 Y	

Injection number, number of injection in build-up phase; Total duration, total treatment duration; I, Investigated group; C, Control group; NSS, Nasal symptom score; MS, Medicine scores; CT, cutaneous test; S, safety; TNC, Titrated nasal challenge; HRQL, health-related quality of life; Der p, Dermatophagoides pteronyssinus; Sal k, Salsola kali; D.G and T.P, D. Glomerata and T. Paniceum.

### Risk of Bias Assessment in Included Studies

**Figure 2:**
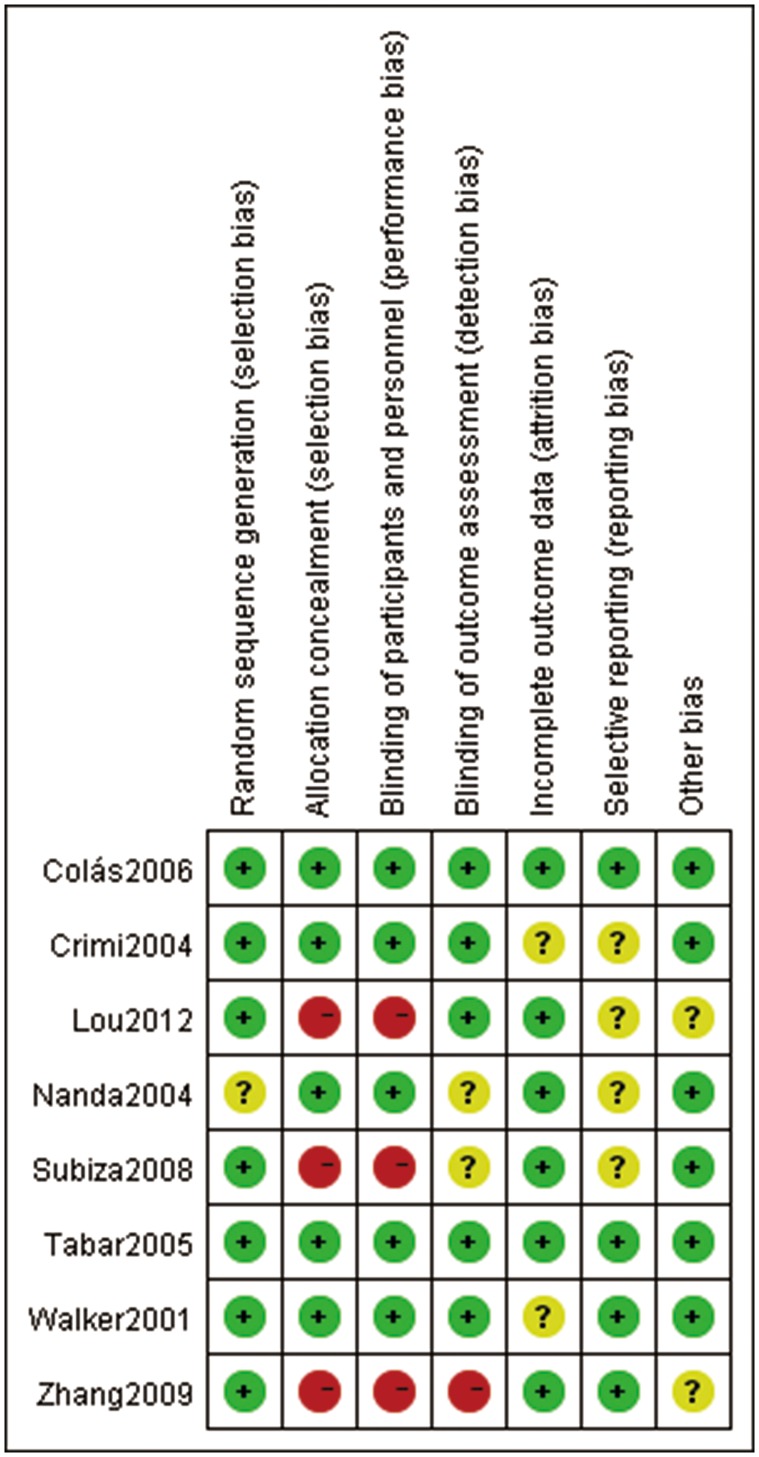
Risk of bias summary: judgements about each risk of bias item for each included study.

Figure 2 provided methodological details for each trial. Among all included trials, randomized sequence were adequately conducted in seven studies [29], [30], [32]–[36], but only one RCT [31] did not adequately describe methods of random sequence generation. Allocation concealment and blinded fashion was clearly stated in five studies [29]–[33]. Blinding of study subjects and investigators was almost universally maintained by use of similar placebo preparations. Three studies [34]–[36] did not conduct allocation concealment and blinded fashion. The outcome measurements about efficacy in this study were subjective, and likely to be influenced by the lack of blinding. Hence, we considered there are some risks in detection bias. The numbers and reasons for withdrawal or dropout were reported in details in all trials. Methods of statistical analysis were well described in all studies.

### Symptom Scores

Symptom reporting remains the most appropriate end point for the study of AR. Six of the included studies reported symptom scores, recorded in patient diaries, as a primary outcome measure [29], [30], [32], [33], [35], [36].

#### Cluster SCIT vs placebo

**Figure 3:**
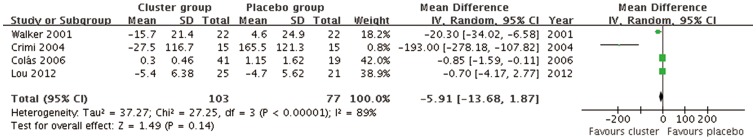
Meta-analysis of the RCTs comparing symptom scores between cluster group and placebo group.

Data on symptom scores were available in four trials [29], [30], [33], [36](n = 180). There was a high degree of heterogeneity between the studies when combined in the meta-analysis (P<0.00001, *I* ^2^ = 89%) with a total of 103 subjects in the cluster group and 77 in the placebo group, which was associated with a non-significant trend in favour of the cluster group, WMD = −5.91 (95% CI, −13.68 to 1.87, P = 0.14; 95% Prediction Interval, −37.24 to 25.42; Figure 3). Sensitivity analysis was performed by sequentially excluding individual studies, but significant heterogeneity between studies was still evident, and the test for overall effect remained not significant.

#### Cluster SCIT vs conventional SCIT

**Figure 4:**
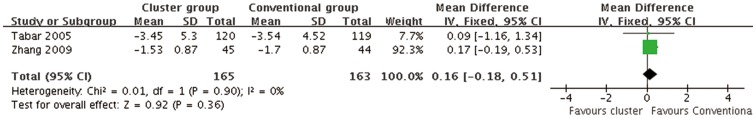
Meta-analysis of the RCTs comparing symptom scores between cluster group and conventional group.

Two RCTs [32], [35] compared cluster SCIT with conventional SCIT(n = 328). In the double-blind study of Tabar et al. [32], 239 adult allergic patients with AR sensitized to *dermatophagoides pteronyssinus* were randomized in two up-dose schedules. 120 patients received a 6-week cluster schedule, whereas 119 patients were randomized to a 12-week conventional one. This trial featured an interesting study design as placebo injections were administered to the“cluster patients”after attaining the maximal dose in weekly intervals until the patients in the conventional group attained their maximal dose at week 12. Though the total duration of the dose-increase phase was reduced by 46% in the cluster group compared with the conventional therapy, the reduction in symptom scores were found 6 weeks earlier in the cluster group than in the conventional group. Our meta-analysis indicated that cluster group and conventional group have a similar reduction in symptom scores (WMD = 0.16, 95% CI −0.18 to 0.51, P = 0.36; P for heterogeneity = 0.90, *I* ^2^ = 0%; Figure 4).

### Medication Scores

Diary scores reflecting concurrent use of anti-allergic medication were reported in 6 studies [29], [30], [32], [33], [35], [36].

#### Cluster SCIT vs placebo

**Figure 5:**
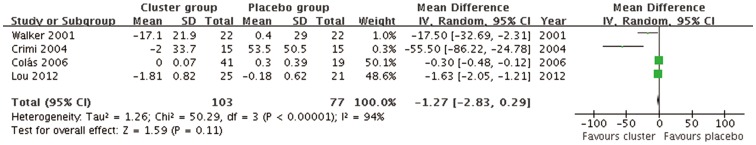
Meta-analysis of the RCTs comparing medication scores between cluster group and placebo group.

Data on medication scores were available in four trials [29], [30], [33], [36](n = 180). The combined WMD and P value was −1.27 (95% CI, −2.83 to 0.29, P = 0.11; 95% Prediction Interval, −7.19 to 4.65). *I* ^2^ was 94%, P<0.00001 indicating significant significantly high level of heterogeneity(Figure 5). Sensitivity analysis was performed by sequentially excluding individual studies, however, significant heterogeneity between studies was still evident, and the result was not materially altered.

#### Cluster SCIT vs conventional SCIT

**Figure 6:**
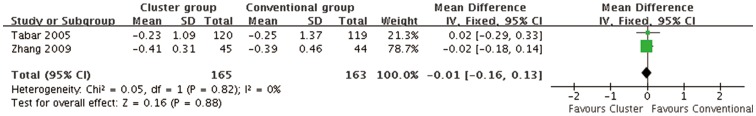
Meta-analysis of the RCTs comparing medication scores between cluster group and conventional group.

Two RCTs [32], [35] compared cluster SCIT with conventional SCIT(n = 328). Pooled results suggested that cluster group and conventional group have a similar reduction in medication scores(WMD = −0.01, 95% CI −0.16 to 0.13, P = 0.88; P for heterogeneity = 0.82, *I* ^2^ = 0%; Figure 6).

### Overall Quality of Life

Three of the included studies reported the Quality of Life Questionnaire [27], [31], [33].

#### Cluster SCIT vs placebo

**Figure 7:**
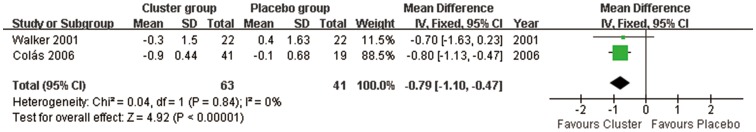
Meta-analysis of the RCTs comparing overall quality of life between cluster group and placebo group.

The meta-analysis of two RCTs [29], [33] revealed that cluster SCIT was superior to placebo in improving overall quality of life.(n = 104; WMD = −0.79, 95% CI, −1.10 to −0.47, P<0.00001; heterogeneity: P = 0.84, *I* ^2^ = 0%; Figure 7).

#### Cluster SCIT vs conventional SCIT

Only one study [35] evaluated quality of life. It showed both cluster and conventional groups obtained satisfactory improvements compared with the baseline in overall quality of life. Therefore, we didn’t perform meta-analysis.

### Adverse Events

Seven studies [29]–[35] reported adverse events in their outcomes. Adverse events were analyzed as local or systemic reactions, and in the build-up phase or maintenance phase. Local reactions were analyzed in two groups according to their need for treatment. Systemic reactions were analyzed according to their severity (grading system) and time of onset (before or after 30 minutes). Grading system followed the European Academy of Allergology and Clinical Immunology Position Paper [37]. Table 2 provided an overview of the adverse events reported in the included studies. Neither fatal events nor systemic reaction grade 3 or 4 were reported in any of the included studies.

**Table 2:** Adverse events.

Study	Build-up phase					Maintenance phase	
	LRNT	LRT	ESRG1	ESRG2	LSR	LR	SR
Walker2001	0	0	0	0	4(I)5(C)	0	3(I)0(C)
Crimi2004	9(I)3(C)	0	0	0	0	3(I)0(C)	0
Nanda2004	0	0	0	1(I)0(C)	0	0	0
Tabar2005	6(I)5(C)	0	0	1(I)2(C)	3(I)2(C)	1(I)1(C)	4(I)3(C)
Colás2006	16(I)10(C)	0	0	4(I)0(C)	12(I)4(C)	0	0
Subiza2008	0	7(I)0(C)	0	0	0	0	0
Zhang2009	11(I)9(C)	0	3(I)4(C)	2(I)2(C)	0	7(I)5(C)	6(I)4(C)
Lou2012	NA	NA	NA	NA	NA	NA	NA

NA, Not available data; LRNT, Local reaction not requiring treatment; LRT, Local reaction requiring treatment;

ESRG1, Early systemic reaction grade 1(<30 minutes); ESRG2, Early systemic reaction grade 2(<30 minutes);

LSR, Late systemic reaction (>30 minutes); LR, Local reaction; SR, Systemic reaction; I,Investigated group; C,Control group.

#### Local adverse reactions (Cluster SCIT vs placebo)

**Figure 8:**
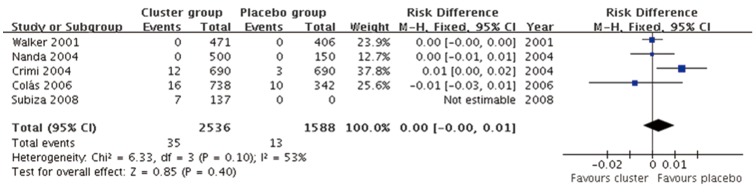
Meta-analysis of the RCTs comparing incidence of local adverse reaction between cluster group and placebo group.

Five studies [29]–[31], [33], [34] data with 2536 cluster SCIT injections and 1588 placebo injections were included. The combined RD was 0.00 (95%CI, −0.00 to 0.01, P = 0.40) indicating no differences in incidence of local adverse reaction between cluster SCIT and placebo(Figure 8), although medium level of heterogeneity was observed (P = 0.10, *I* ^2^ = 53%).

#### Local adverse reactions (Cluster SCIT vs conventional SCIT)

**Figure 9:**
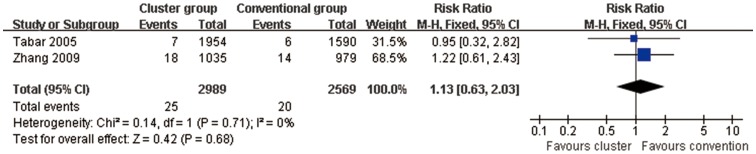
Meta-analysis of the RCTs comparing incidence of local adverse reaction between cluster group and conventional group.

For 2 studies [32], [35] comparing cluster group with conventional group combined RR was 1.13 (95% CI, 0.63 to 2.03, P = 0.68) indicating no differences in incidence of local adverse reaction(Figure 9). *I* ^2^ was 0%, P = 0.71 indicating lack of heterogeneity.

#### Systemic adverse reactions (Cluster SCIT vs placebo)

**Figure 10:**
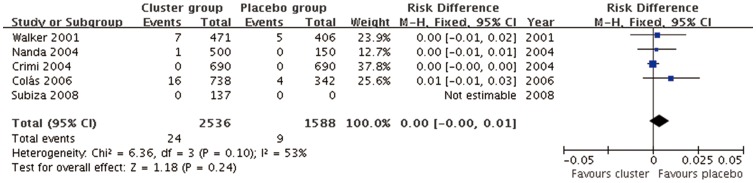
Meta-analysis of the RCTs comparing incidence of systemic adverse reaction between cluster group and placebo group.

The aggregated results of 5 studies [29]–[31], [33], [34] suggested that no differences existed in the incidence of systemic adverse reaction between cluster group and placebo group (RD = 0.00, 95% CI, −0.00 to 0.01, P = 0.24; Figure 10), yet medium level of heterogeneity was present (P = 0.10, *I* ^2^ = 53%).

#### Systemic adverse reactions (Cluster SCIT vs conventional SCIT)

**Figure 11:**
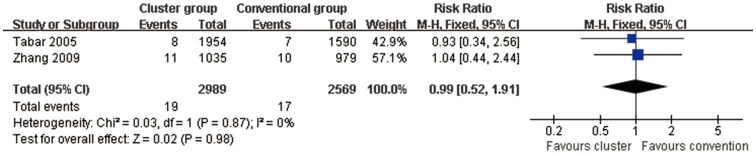
Meta-analysis of the RCTs comparing incidence of systemic adverse reaction between cluster group and conventional group.

The incidence of systemic adverse reaction were not different between patients of the cluster group and conventional group (2RCTs; RR = 0.99, 95% CI, 0.52 to 1.91, P = 0.98; Figure 11). There was no evidence of heterogeneity for these outcomes (P = 0.87, *I* ^2^ = 0%).

### Publication Bias

There was no evidence of significant publication bias by inspection of the funnel plots.

## Discussion

In the present study, we have performed a comprehensive systematic review of the efficacy and safety of cluster SCIT in AR patients.

This systematic review found eight RCTs which satisfied our inclusion criteria. Scores representing symptom severity and scores quantifying concurrent medication use were recorded in six of the included studies. Our meta-analysis showed that cluster SCIT have similar effect in reduction of both rhinitis symptoms and the requirement for anti-allergic medication compared with conventional SCIT, but when comparing cluster SCIT with placebo, no statistic significance were found in reduction of symptom scores or medication scores. Some caution is required in this interpretation as there was significant heterogeneity between studies. This high degree of heterogeneity resulted predominantly from the wide variety of scoring systems used across studies, although it is in part compensated for by use of the weighted mean difference in the meta-analyses. Furthermore, it is difficult to know what constitutes a clinically important difference on these scales; the range of symptom scores used potentially also threatens the appropriateness of combining data in a meta-analysis. We consequently feel these combining results were insufficient for us to draw certain conclusions, and standardized scoring systems are needed.

The Rhinoconjunctivitis Quality of Life Questionnaire (RQLQ) [38], assessing the impact of symptoms on work, school, and leisure activities, represent an important, often overlooked outcome measure in immunotherapy studies. Data relating to RQLQ in 3 included studies were analyzed, and point to the superior efficacy of cluster SCIT in two studies compared to placebo and in one study compared to conventional SCIT.

Some of the observed variability in treatment effects may be explained by variable responses to treatment according to the type of allergen used, the quality of allergen vaccines used, the geographical background of participants in these trials, the age of subjects studied or the duration of treatment given. As these may be significant factors when selecting suitable individuals for future treatment. In this study, we also found the cluster protocols in all these studies finally reached the dose in build-up phase (maintenance dose) which ranged from 4.8 to 22.5 µg. This is important because therapeutic efficacy requires high allergen doses (Low dose immunotherapy is usually ineffective [4], therefore therapeutic efficacy correlates with an optimal maintenance dose in the range of 5 to 20 µg of major allergen per injection for a number of primary allergens).

Principles of accelerated schedules in SCIT have been described first by Freeman in the thirties of the nineteenth century as “intensive desensitization” [39]. However, these schedules have not been widely used in Europe and in the US, likely to safety concerns. The safety of cluster SCIT has always been the important subject of this treatment. To our knowledge, this study is the first meta-analysis to evaluate the safety of cluster SCIT in AR patients. The pooled results from the meta-analysis showed that no differences existed in the incidence of either local adverse reaction or systemic adverse reaction between the cluster group and control group.

Furthermore, we found local symptoms were readily reversible without treatment in the majority of these studies. In a few cases, local reactions completely recovered after appropriate treatment. We didn’t find systemic reaction of grade 3 or 4 and fatal events that was reported in any of the included studies. All systemic reactions reported were mild (grade 1 or 2) rhinoconjunctivitis, mild wheezing, urticaria, otic pruritus, itching palms/soles, edema of the eyelid, and so on. It was reported that all subjects fully recovered after treatment and none dropped out following these reactions.

Further studies should focus on the three following points. First, it is necessary to standardize a cluster SCIT schedule protocol (further consistency regarding type of vaccine, dosage, frequency, and duration of administration) since great variability exists in the literature. Next, the standardized scoring systems, as mentioned above, should be used to evaluate the efficacy of cluster SCIT. Finally, there was only one accepted study of cluster SCIT that was conducted exclusively in children in this systematic review, raising the question whether cluster SCIT is be efficacious and safe to patients younger than 18 years of age. More RCTs are required to perform to answer this question.

## Conclusion

Based on the current limited evidence, we still could not conclude affirmatively that cluster SCIT was a safe and efficacious option for the treatment of AR patients. Further large-scale, well-designed RCTs on this topic are still needed. Taken the risk-benefit balance into consideration, cluster SCIT might turn these schedules to become an interesting SIT option “ready for the practical use” in the future.

## Supporting Information

Checklist S1 PRISMA 2009 Checklist.
(DOC)
